# PPFIA4 promotes castration-resistant prostate cancer by enhancing mitochondrial metabolism through MTHFD2

**DOI:** 10.1186/s13046-022-02331-3

**Published:** 2022-04-05

**Authors:** Ru Zhao, Tingting Feng, Lin Gao, Feifei Sun, Qianqian Zhou, Xin Wang, Junmei Liu, Wenbo Zhang, Meng Wang, Xueting Xiong, Wenqiao Jia, Weiwen Chen, Lin Wang, Bo Han

**Affiliations:** 1 The Key Laboratory of Experimental Teratology, Ministry of Education and Department of Pathology, School of Basic Medical Sciences, Cheeloo College of Medicine, Shandong University, Jinan, Shandong China; 2 Department of Biochemistry and Molecular Biology, School of Basic Medical Sciences, Cheeloo College of Medicine, Shandong University, Jinan, Shandong China; 3 Department of Molecular Genetics, University of Toronto, Toronto, ON Canada; 4 Department of Health Management CenterQilu Hospital, Cheeloo College of Medicine, Shandong University, Jinan, Shandong China; 5 Department of Oncology, The First Affiliated Hospital of Shandong First Medical University; Biomedical Sciences College & Shandong Medicinal Biotechnology Centre, Key lab for Biotech-Drugs of National Health Commission, Key Lab for Rare & Uncommon Diseases of Shandong Province, Shandong First Medical University & Shandong Academy of Medical Sciences, Jinan, Shandong China; 6 Department of Pathology, Qilu Hospital, Cheeloo College of Medicine, Shandong University, Jinan, Shandong China

**Keywords:** CRPC, mitochondrial function, PPFIA4, MTHFD2

## Abstract

**Background:**

The development of castration-resistant prostate cancer (CRPC) remains a major obstacle in the treatment of prostate cancer (PCa). Dysregulated mitochondrial function has been linked to the initiation and progression of diverse human cancers. Deciphering the novel molecular mechanisms underlying mitochondrial function may provide important insights for developing novel therapeutics for CRPC.

**Methods:**

We investigate the expression of the protein tyrosine phosphatase receptor type F polypeptide interacting protein alpha 4 (PPFIA4) using public datasets and tumor specimens from PCa cases by immunohistochemistry. Gain- and loss-of-function studies are performed in PCa cell lines and mouse models of subcutaneous xenograft to characterize the role of PPFIA4 in CRPC. Gene expression regulation is evaluated by a series of molecular and biochemical experiments in PCa cell lines. The therapeutic effects of methylenetetrahydrofolate dehydrogenase 2 (MTHFD2) inhibitor combined enzalutamide are assessed using *in vitro* functional assays and *in vivo* mouse models.

**Results:**

We show that the increase of PPFIA4 exacerbates aggressive phenotype resembling CRPC. A fraction of PPFIA4 localizes to mitochondria and interacts with MTHFD2, a key enzyme for one-carbon metabolism. Androgen deprivation increases the translocation of PPFIA4 into mitochondria and increases the interaction between PPFIA4 and MTHFD2, which result in the elevation of tyrosine phosphorylated MTHFD2. Consequently, the levels of NADPH synthesis increase, resulting in protection against androgen deprivation-induced mitochondrial dysfunction, as well as promotion of tumor growth. Clinically, PPFIA4 expression is significantly increased in CRPC tissues compared with localized PCa ones. Importantly, an MTHFD2 inhibitor, DS18561882, combined with enzalutamide can significantly inhibit CRPC cell proliferation *in vitro* and tumor growth *in vivo*.

**Conclusion:**

Overall, our findings reveal a PPFIA4-MTHFD2 complex in mitochondria that links androgen deprivation to mitochondrial metabolism and mitochondrial dysfunction, which suggest a potential strategy to inhibit CRPC progression.

**Supplementary Information:**

The online version contains supplementary material available at 10.1186/s13046-022-02331-3.

## Background

Onset of castration-resistance prostate cancer (CRPC) after long-term androgen deprivation therapy (ADT) remains a major obstacle in the treatment of prostate cancer (PCa) [1]. While androgen receptor (AR) signaling is critically required for CRPC, AR-independent signaling pathways, such as glucocorticoid receptor activation, immune-mediated resistance, and neuroendocrine differentiation could also contribute to CRPC progression [2].

Mitochondria are bioenergetic and biosynthetic organelles that coordinate cellular adaptation to stressors, such as nutrient deprivation, oxidative stress, DNA damage and endoplasmic reticulum stress [3]. In addition to ATP, mitochondria produce metabolic precursors for macromolecules such as lipids, proteins, DNA and RNA to meet the need of cell growth and proliferation [4]. Mitochondria also generate metabolic by-products, such as reactive oxygen species (ROS) and ammonia, and they possess mechanisms to clear or utilize waste products [5]. Importantly, the metabolic reprogramming in cancer cells is mechanistically linked to oncogenic signals. Targeting mitochondria as a cancer therapeutic strategy has attracted much attention in the recent years. A growing body of evidence suggests that mitochondria are associated with PCa progression due to the reactivation of mitochondrial oxidative phosphorylation [6–8]. Molecular links are also observed between androgen signaling, mitochondrial morphology, and metabolic reprogramming in PCa [9, 10]. Furthermore, mitochondrion-rich phenotype is strongly linked to poor prognosis in ERG negative PCa patients [11]. It has been shown that androgen deprivation could trigger impairment of mitochondria membrane in PCa cells [12, 13], and Ca^2+^ overload or oxidative stress induced by the impairment of mitochondrial metabolic function and structural dynamics can lead to CRPC cell death [14].. These findings indicate that enhanced mitochondrial activity, particularly in androgen deprivation condition, might contribute to the ADT failure and CRPC progression.

The evolutionary conserved liprin family, including α and β multidomain adaptor proteins, is involved in the organization of synaptic vesicles and neurotransmitter receptors [15–17]. Liprin-α4 (encoded by PPFIA4), a member of liprin-α family, has been found in various multiprotein complexes [18, 19]. It has been reported as a new potential therapeutic target for refractory pancreatic cancer [20], small cell lung cancer [21], and clear cell renal cell cancer [18]. Additionally, PPFIA4 has been reported in several studies to be potentially associated with aberrant metabolic processes [22, 23]. In the current study, we demonstrate that PPFIA4 expression is increased in PCa cells under androgen-deprived condition and in CRPC tissues. PPFIA4 can promote PCa progression, especially the occurrence of CRPC. A fraction of PPFIA4 localizes in mitochondria and interacts with MTHFD2 in response to androgen deprived challenge. MTHFD2 is generally regarded as the enzyme responsible for mitochondrial NADPH production to overcome oxidative stress and maintain redox homeostasis in tumor cells [24], and MTHFD2 deficiency can induce mitochondrial dysfunction [25]. Next, we investigate whether androgen deprivation regulates PPFIA4 expression and how PPFIA4 regulates MTHFD2-mediated mitochondrial function to promote CRPC progression.

## Methods

### Patients and tissue specimens

A total of three tissue microarrays were constructed representing 132 clinically localized PCa patients who underwent radical prostatectomy, and 25 PCa patients with CRPC treated by transurethral resection of the prostate between 2012 and 2015 at Qilu Hospital of Shandong University (Jinan, China). This study was conducted following the International Ethical Guidelines for Biomedical Research Involving Human Subjects. This study protocol was approved by Shandong University Medical Research Ethics Committee according to the Declaration of Helsinki (Document No. ECSBMSSDU2021-1-61).

### Immunohistochemistry (IHC)

IHC and scoring analysis of the staining intensity were carried out as described previously [26]. Antigen retrieving was performed in Tris (pH 6.0) in a pressure cooker for 10 minutes. The tissue slides were incubated with the indicated primary antibodies overnight at 4°C. Primary antibodies used in this study are anti-PPFIA4 (1:50, HPA053419, Sigma) and anti-Ki67 (ZA-0502, Zsbio). IHC intensity was assessed as described in the previous article [27]. For assessment of intensity, each field was graded semi-quantitatively on tree-tier scale (0 = none staining, 1 = weak staining, 2 = moderate staining, 3 = strong staining). For analysis, we combined both negative and weakly PPFIA4 positive tumors into one group and moderately and strongly PPFIA4 positive PCa into the other.

### Cell culture and drug treatments

LNCaP, C4-2B, VCaP, PC3, and HEK293T cells were purchased from American Type Culture Collection (ATCC; Manassas, VA, USA) and cultured following ATCC’s instructions. All cell lines used in this study were tested for negative mycoplasma contamination and authenticated by short tandem repeat assays. For the androgen deprivation, LNCaP cells were hormone-starved in the Phenol Red-free medium containing 10% charcoal-stripped fetal bovine serum (CSS; Hyclone, Logan, UT, USA). DS18561882, N-acetyl-L-cysteine (NAC), PP2 and enzalutamide were purchased from MedChemExpress.

### RNA isolation and quantitative real-time PCR (qRT-PCR) analysis

Total RNA was extracted using the TRIzol reagent (Vazyme Biotech, R401-01 regent kit) and reverse-transcribed with ReverTra Ace qPCR RT kit (Toyobo, PCR-311) according to the manufacturer’s protocol. qRT-PCR was performed using the SYBR Green mix (Toyobo, QPK-201). The primer sequences used are listed in Supplementary Table 1.

### Western blotting and immunoprecipitation

Western blotting and immunoprecipitation assays were performed as previously described [28]. To determine the subcellular localization of PPFIA4, cytoplasmic and mitochondrial proteins were extracted using the Cell Mitochondria Isolation Kit (C3601, Beyotime Biotechnology, Jiangsu, China) following the manufacturer’s instructions. The information of antibodies is summarized in Supplementary Table 2.

### Transient transfection and viral transduction

Human PPFIA4 and MTHFD2 plasmids were purchased from Sangon Biotech (Shanghai, China). All siRNA and negative controls were designed and synthesized by Ribobio (Guangzhou, China). The target sequences are listed in Supplementary Table 3. To avoid off-target effects, co-transfection of two RNAs with better interference efficiency was performed. Lipofectamine 3000 (Invitrogen, Carlsbad, CA) was used for transfection following the manufacturer’s instructions. Human Lenti-PPFIA4-EGFP and its control Lenti-EGFP were obtained from Genecopoeia. To acquire a stable expression cell line, single-cell clonal isolates were selected by puromycin.

### Mitochondrial respiration

An extracellular flux analyzer (XF96: Seahorse Biosciences Agilent, Santa Clara, CA, USA) was used to analyze mitochondrial function as described previously [29]. Briefly, LNCaP or C4-2B cells were plated in each well of a Seahorse XFe96 cell culture plate. Oxygen consumption rate (OCR) was measured under basal conditions and after addition of oligomycin (1 μM), carbonyl cyanide-4-(trifluoromethoxy) phenylhydrazone (FCCP) (1 μM), and antimycin (0.5 μM).

### Xenograft studies in nude mice

4-6-week-old male nude mice were purchased from Weitonglihua Biotechnology (Beijing, China). The CRPC model was studied as previously described [26]. 2 × 10^6^ VCaP or 1 × 10^7^ LNCaP cells with overexpression of control vector or PPFIA4 and 1 × 10^7^ C4-2B cells expressing control shRNA (shEGFP) or shPPFIA4 were suspended in 100 μl of PBS with 50% Matrigel and injected subcutaneously into the mice. Tumor volume (length × width^2^ × 0.5) was measured twice a week. Tumor-bearing mice were castrated when the tumors were approximately 300 mm^3^ in size. Then the mice were randomized and treated with DS18561882 (100mg/kg, p.o.) or vehicle control (PBS, p.o.) once daily (*n =* 5/group). For enzalutamide and DS18561882 combination treatment, mice were randomly selected to enzalutamide (10 mg/kg, p.o.) plus either DS18561882 (100 mg/kg, p.o.) or PBS (p.o.) once daily (*n =* 5/group). The experimental protocols were performed following the Ethical Animal Care and Use Committee of Shandong University (Document No. ECSBMSSDU2021-2-126).

### RNA sequencing (RNA-seq) and bioinformatics analysis

We performed RNA-seq analyses (Kangcheng, Shanghai, China) to compare the mRNA expression profiles between control (NC) and PPFIA4 knockdown (siPPFIA4) C4-2B cells. The expressed genes were analyzed for enrichment of biological themes using Gene Set Enrichment Analysis (GSEA) (http://software.broadinstitute.org/gsea/index.jsp). Datasets of GSE35988, GSE68882, GSE6919, GSE21034, GSE3325 and GSE32269 were downloaded from the Gene Expression Omnibus (GEO) database (http://www.ncbi.nlm.nih.gov/geo). GEPIA (http://gepia.cancer-pku.cn/detail.php) was used to analyze the relationship between PPFIA4 expression and disease-free survival of PCa cases.

### Statistical analysis

Statistical analysis was carried out using GraphPad Prism 7 or the SPSS 20.0 software. The two-tailed unpaired t-test was used to calculate statistical significance between the two groups. Survival information was verified by Kaplan-Meier analysis and compared using the log-rank test. All experiments *in vitro* were performed in biological triplicate. All results are presented as the mean and the standard error of the mean. The tumor growth was analyzed by ANOVA. *P* values considered to be significant as follows: *p < 0.05; **p < 0.01; ***p < 0.001 and ****p < 0.0001.

## Results

### PPFIA4 is androgen-responsive and upregulated in CRPC

**Fig. 1:**
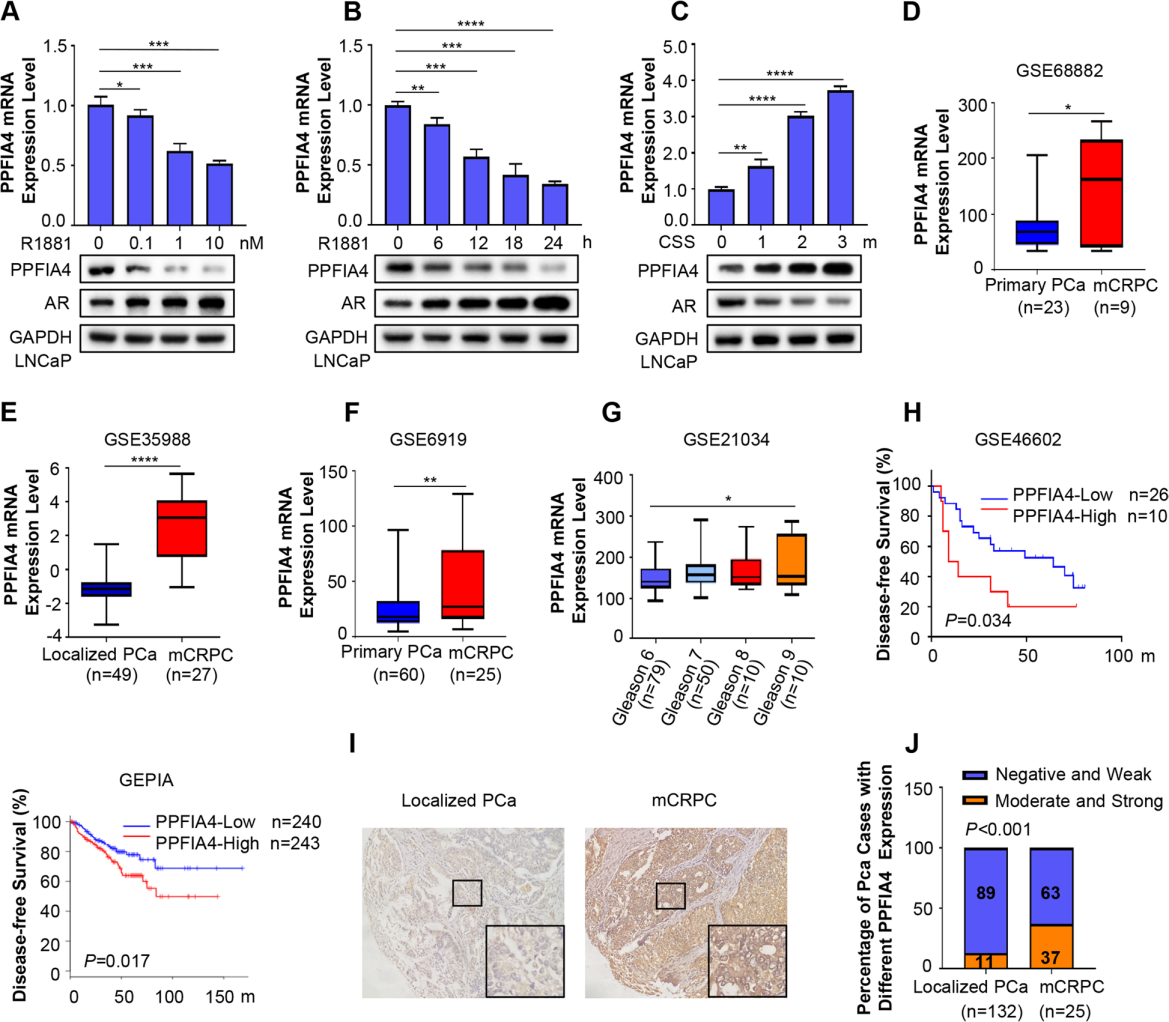
The expression of PPFIA4 is upregulated in CRPC. **A-B**. The mRNA and protein levels of PPFIA4 in LNCaP cells determined by qRT-PCR and western blotting. LNCaP cells were treated with 0.1, 1, and 10 nM R1881 for 24 hours (**A**) or treated with 1 nM R1881 at the indicated time points (**B**). Western blotting was performed with the indicated antibodies. GAPDH was used as a loading control. *p < 0.05, **p < 0.01, ***p < 0.001, ****p < 0.0001 based on the Student’s *t*-test. h, hours. **C**. The mRNA and protein levels of PPFIA4 in LNCaP cells with prolonged androgen-deprived treatment (1, 2, and 3 months) determined by qRT-PCR and western blotting. Western blotting was performed with the indicated antibodies. GAPDH was used as a loading control. **p < 0.01, ****p < 0.0001 based on the Student’s *t*-test. CSS, charcoal-stripped serum. m, months. **D-G**. PPFIA4 expression in metastatic CRPC (mCRPC) tissues compared to primary localized PCa samples and in PCa patients with different Gleason scores in public datasets (GSE68882, GSE35988, GSE6919 and GSE21034). *p < 0.05, **p < 0.01, ****p < 0.0001. H. Kaplan–Meier survival analysis of PCa cases from GSE46602 (*p* = 0.034, Log-rank test) and GEPIA prostate cohort (*p* = 0.017, Log-rank test). m, months. I. Representative IHC images of PPFIA4 in localized PCa and mCRPC tissues. Magnified images from the regions marked by rectangles were showed in the bottom. J. Percentages of different PPFIA4 expression (out of 100%) distributed in 157 PCa cases in Qilu Hospital with primary localized PCa or CRPC

Androgen-AR signaling is critical for PCa cells. In the current study, we first ask whether PPFIA4 is responsive to androgen. As shown in Fig. 1A-B, PPFIA4 decreased in a dose- and time-dependent manner at both mRNA and protein levels when LNCaP cells were treated with synthetic androgen R1881. By contrast, androgen ablation significantly increased both PPFIA4 mRNA and protein levels in LNCaP cells (Fig. 1C). The protein levels of AR (Fig. 1A-C) and PSA (Supplementary Fig. S1A- S1C) increased in response to R1881 treatment but decreased upon androgen deprivation. These data showed that the expression levels of PPFIA4 were inversely correlated with AR and PSA. We next analyzed publicly available human PCa datasets for PPFIA4 expression. Detailed bioinformatics analysis revealed that PPFIA4 expression was significantly increased in CRPC compared with primary localized PCa in GSE68882, GSE35988, and GSE6919 datasets (Fig. 1D-F). Of note, as shown in Fig. 1G, PCa cases from public dataset GSE21034 assigned to high Gleason scores showed increased PPFIA4 expression. We also found that PCa patients with high PPFIA4 mRNA expression displayed worse disease-free survival in the GSE46602 dataset and GEPIA (Fig. 1H). These results are consistent with previous reports that PPFIA4 acts as a potential prognostic factor in PCa [30, 31] and indicate that high PPFIA4 expression is associated with PCa progression.

To further confirm these findings and to measure PPFIA4 protein expression in PCa patients, we investigated a cohort of 157 PCa patient samples (Qilu cohort) from our hospital by IHC assay. Representative IHC images are shown in Fig. 1I. Among 132 patients with clinically localized PCa, 117 (89%) showed negative or weak staining (scores 0 and 1), and only 15 (11%) had moderate or strong staining (scores 2 and 3). In contrast, among the 25 PCa patients with CRPC, 9 (37%) cases showed moderate to strong staining, whereas only 16 (63%) showed negative or weak staining of PPFIA4 (Fig. 1J). Collectively, these results indicate that the increased PPFIA4 expression is associated with CRPC progression.

### PPFIA4 is an AR-repressed gene in PCa cells

**Fig. 2:**
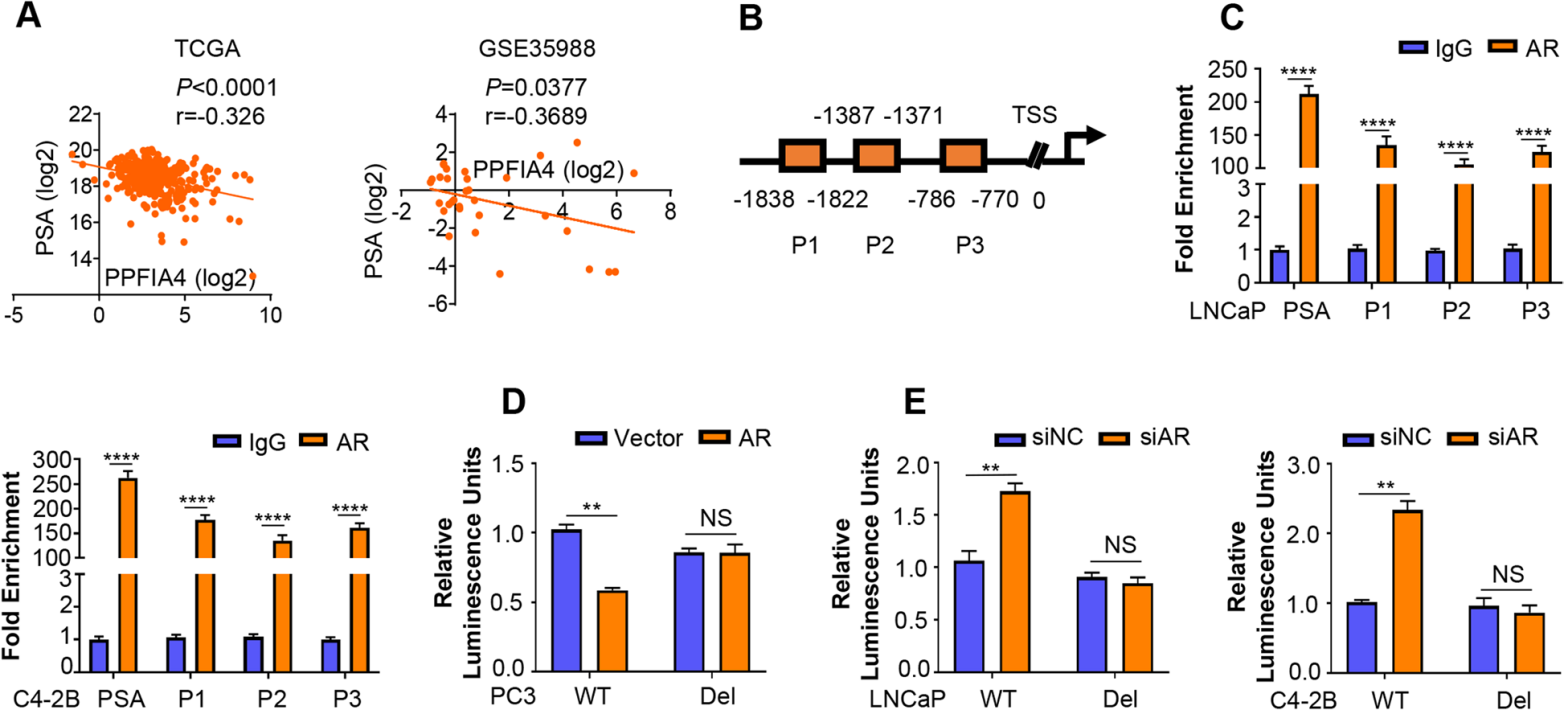
PPFIA4 is an AR-repressed gene. **A**. Pearson correlation of mRNA expression between PPFIA4 and AR-targeted gene PSA. Transcriptomic data from TCGA and GSE35988 clinical prostate cancer cohort was used to perform correlation analysis. **B**. Schematic of three putative AR binding sites on the PPFIA4 gene promoter. According to motif analysis in JASPAR database, primers were designed as P1 (-1838 to -1822), P2 (-1387 to -1371) and P3 (-786 to -770) based on their distances to the transcription start site (TSS) of PPFIA4 gene. **C**. ChIP analysis was conducted in LNCaP and C4-2B cells to validate AR enrichment on PPFIA4 promoter. Purified rabbit IgG was used as negative control. Primers flanking the AR binding site on the PSA gene promoter were used as positive controls. ****p < 0.0001 based on the Student’s *t*-test. **D-E**. Luciferase reporter assays were performed in PC3 cells transfected with empty vector or PPFIA4 overexpression plasmid (**D**) and in LNCaP or C4-2B cells transfected with negative control (siNC) or AR siRNA (siAR). Luciferase reporters containing either wild-type or AR binding sites deletion in PPFIA4 promoter were constructed. Luciferase activities were measured 48 hours after transfection. **p < 0.01 based on the Student’s *t*-test. NS, no significance

To determine whether AR regulates PPFIA4 or not, we examined the relationship between PPFIA4 and the AR target gene PSA. As shown in **Fig.** **2****A,** PPFIA4 mRNA expression of PCa samples in multiple clinical cohorts from public datasets (TCGA and GSE35988) tend to be inversely correlated with PSA mRNA expression. Consistently, our results also showed that PPFIA4 was inversely correlated with the protein levels of AR and PSA in LNCaP cells under R1881 or androgen deprivation treatment (Fig. 1A-C; Supplementary Fig. S1A-C). In addition, we performed motif analysis (http://jaspar.genereg.net/) and found three potential AR-binding sites named as P1 (-1838 to -1822), P2 (-1387 to -1371) and P3 (-786 to -770) at the promoter of the PPFIA4 gene (Fig. 2B). Site-specific ChIP analysis showed the recruitment of AR was enriched to the above three sites in the PPFIA4 promoter of LNCaP and C4-2B cells (Fig. 2C). Our luciferase reporter assay illustrated that PC3 cells with exogenous AR introduction suppressed PPFIA4 wild type but not the mutant (PPFIA4-del) promoter activity (Fig. 2D). By contrast, AR knockdown activated PPFIA4 wild type promoter activity in LNCaP and C4-2B cells (Fig. 2E). Taken together, our data demonstrated AR as a transcriptional repressor of PPFIA4.

Next, to evaluate the effect of PPFIA4 on AR expression and activity, two independent siRNAs against PPFIA4 were synthesized and their silencing efficiency was confirmed by western blotting (Supplementary Fig. S1D). As shown in Supplementary Fig. S1E-G, siRNA knockdown or ectopic overexpression of PPFIA4 did not alter AR protein expression and activity in LNCaP cells. In addition, no physical interaction between PPFIA4 and AR was detected by co-immunoprecipitation (Co-IP) in LNCaP cells (Supplementary Fig. S1H).

### PPFIA4 overexpression renders PCa cells with a CRPC phenotype

**Fig. 3:**
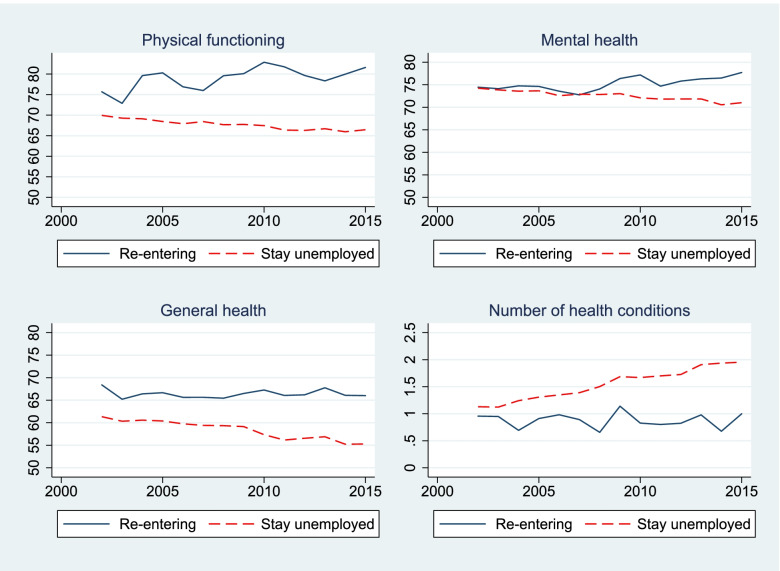
PPFIA4 promotes PCa cell growth *in vitro* and *in vivo*. **A-D**. Cell proliferation was assessed by cell counts and colony formation assays in C4-2B cells transfected with siNC or PPFIA4 siRNA (siPPFIA4) (**A-B**) or in LNCaP cells transfected with empty vector or PPFIA4 overexpression plasmid with or without androgen deprivation (**C-D**). Cell number was counted at the indicated time points and all the numbers were normalized to day 0 (**A, C**). Data shown are presented as means ± SEM of triplicate wells and are representative of at least three replicate experiments. Representative images of colony formation assays are shown in the left panel and quantitative analysis is shown in the right panel (**B, D**). **p < 0.01, ***p < 0.001, ****p < 0.0001 based on the Student’s *t*-test. d, days. FBS, fatal bovine serum. CSS, charcoal-stripped serum. E-H. EdU assays (**E-F**) and cell apoptosis assays (G-H) were performed in indicated PCa cells with PPFIA4 knockdown or overexpression. Representative images are shown in the left panel and quantitative analysis is shown in the right panel. * *p* < 0.05. **p < 0.01, ***p < 0.001, ****p < 0.0001. FBS, fatal bovine serum. CSS, charcoal-stripped serum. I-L. The tumor volume was measured in nude mice with castration as the indicated treatment. LNCaP cells with stable overexpression of PPFIA4 or C4-2B cells with stable knockdown of PPFIA4, as well as their parental controls, were subcutaneously injected into nude mice (*n =* 5/group). The tumor volume was measured twice every week after nude mice were castrated (I, K). Tumors were isolated from mice, photographed, and weighed at the endpoint (J, L). ***p < 0.001

We subsequently investigated the functional role of PPFIA4 in PCa cells. PPFIA4 knockdown significantly inhibited cell proliferation and colony formation in C4-2B and PC3 cells (Fig. 3A-B; Supplementary Fig. S2A-B). In contrast, PPFIA4 overexpression overcame ADT-induced growth arrest and promoted the proliferation and colony formation of LNCaP cells (Fig. 3C-D). EdU incorporation assays further confirmed these findings from cell growth curve and colony formation in C4-2B and LNCaP cells (Fig. 3E-F). In addition, PPFIA4 knockdown increased the apoptotic rates of C4-2B and PC3 cells (Fig. 3G; Supplementary Fig. S2C), while PPFIA4 overexpression reduced the apoptotic rates of LNCaP cells (Fig. 3H). Importantly, LNCaP-PPFIA4-derived tumors (948±114.8 mm^3^) grew more rapidly than their parental controls (415.4±91.6 mm^3^) under castration condition (Fig. 3I-J). In contrast, PPFIA4 depletion in C4-2B xenografts grown in castrated nude mice resulted in delayed tumor progression, with the mean tumor volume at 620±105.2 mm^3^ in C4–2B-shPPFIA4 xenografts but 1363±268.5mm^3^ in the control group (Fig. 3K-L). These results suggest that PPFIA4 promotes PCa cell proliferation and tumor progression in castrated xenograft models.

### PPFIA4 enhances mitochondrial function

**Fig. 4:**
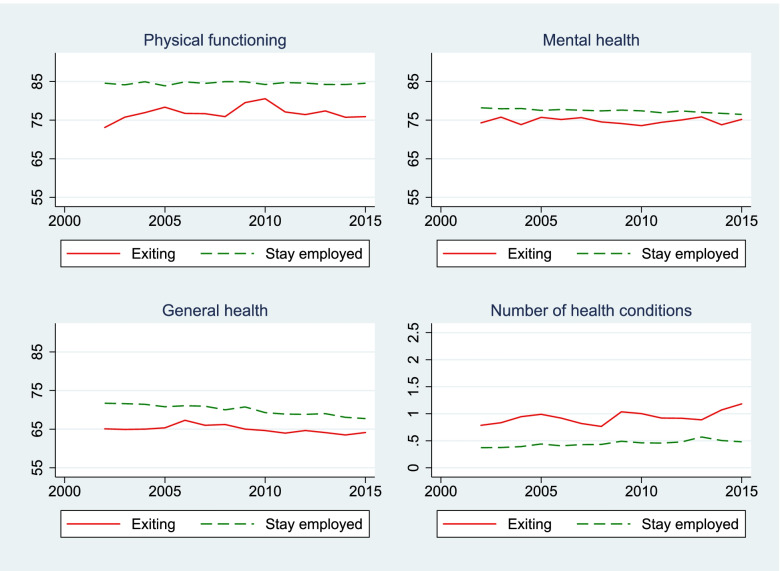
A fraction of PPFIA4 localizes in the mitochondria and confers increased mitochondrial function. **A**. KEGG pathway gene set enrichment analysis of downregulated genes in C4-2B cells with PPFIA4 knockdown. C4-2B cells were transfected with siNC or siPPFIA4 for 48 hours. Total RNA was then extracted and used to perform RNA-Seq analyses. KEGG pathway gene set enrichment analysis was carried out to examine the pathway enrichment. **B**. Organelle localization analysis of PPFIA4 interacting proteins by Web Gestalt (http://www.webgestalt.org/). C4-2B cell lysates were immunoprecipitated with PPFIA4 antibody and resolved by SDS–PAGE. Liquid chromatography-tandem mass spectrometry analysis was performed to identify its interacting proteins. **C**. Localization of PPFIA4 (green) in LNCaP cell mitochondria (red) was verified by immunofluorescent staining with or without androgen deprivation. Representative images are shown with a 10-μm scale bar. Magnified images from the regions marked by rectangles in the top panel was showed in the bottom panel. Mito, mitochondria. FBS, fatal bovine serum. CSS, charcoal-stripped serum. **D**. The protein levels of PPFIA4 in total cell lysates (Total), cytosolic fraction (Cyto), and mitochondrial fraction (Mito) were analyzed by western blotting in LNCaP cells with or without androgen deprivation. COXIV and tubulin were used as mitochondrial and cytosolic markers. **E-F**. Measurement of OCR in indicated PCa cells with PPFIA4 overexpression or knockdown and treated with or without androgen deprivation and enzalutamide (10 μM). Representative recording of OCR during extracellular flow analysis (“Seahorse”) is shown in the up panel, and quantitative analysis of the calculated basal and maximum respiratory rates, ATP production rate and spare respiratory capacity are shown in the bottom panel. *p < 0.05, **p < 0.01, ***p < 0.001, ****p < 0.0001. m, minutes. FBS, fatal bovine serum. CSS, charcoal-stripped serum. ENZ, enzalutamide. **G-L**. Measurement of membrane potentials, NADPH/ NADP^+^ ratio, and ROS levels in LNCaP (G-I) and C4-2B (J-L) cells with PPFIA4 overexpression or knockdown and treated with or without androgen deprivation. **p < 0.01, ***p < 0.001, ***p < 0.001, ****p < 0.0001. FBS, fatal bovine serum. CSS, charcoal-stripped serum. **M**. Cell proliferation was measured by cell counts in C4-2B cells with PPFIA4 knockdown and treated with or without ROS scavenger NAC (5 nM). Cell number was counted at the indicated time points and all the numbers were normalized to day 0. All error bars represent the SD of at least three replicates from three independent experiments. **p < 0.01, ***p < 0.001. d, days

To explore the molecular mechanism by which PPFAI4 promotes the aggressive phenotype of PCa cells, we performed RNA-seq in C4-2B cells with or without PPFIA4 expression. Subsequent Kyoto Encyclopedia of Genes and Genomes (KEGG) analysis showed that the dysregulated genes induced by PPFIA4 knockdown were mostly enriched in cellular metabolism (Fig. 4A). Immunoprecipitation coupled with mass spectrometry were then performed to profile the interactome of PPFIA4 in C4-2B cells. Intriguingly, multiple components of mitochondria were identified as its potential interactors (Fig. 4B). Subsequent immunofluorescent staining and western blotting analysis showed that, besides cytoplasmic compartment, a fraction of PPFIA4 was localized to the mitochondria in LNCaP and C4-2B cells (Fig. 4C-D; Supplementary Fig. S3A). Furthermore, there was an increase of PPFIA4 expression in mitochondria when LNCaP cells were challenged by ADT (Fig. 4C-D). These data indicate that a fraction of PPFIA4 localizes to the mitochondria responsive to ADT and PPFIA4 may be involved in mitochondrial function.

Previously, Audet-Walsh, É., *et al.* demonstrated that mitochondrial respiration increased when LNCaP cells were challenged by sustainable exposure of androgen [32]. However, little is known about the mitochondrial changes during androgen deprivation. Our studies showed that the morphology of mitochondrion became irregular (Supplementary Fig. S3B) and the mitochondrial OCR decreased when LNCaP cells were deprived of androgen (Fig. 4E). To further validate the relation between ADT and mitochondrion, GSEA was performed in microarray data from LNCaP cells with or without androgen deprivation. The data showed that the enrichment of gene sets was functionally associated with mitochondrial protein complexes, oxidative phosphorylation, ATP synthesis, and oxidoreductase complex (Supplementary Fig. S3C). Collectively, these results suggest that ADT disrupt mitochondrial homeostasis and induce mitochondrial dysfunction in PCa cells.

To explore the effects of PPFIA4 on mitochondrial function, we measured mitochondrial respiration in PCa cells. As shown in Fig. 4E-F, PPFIA4 overexpression significantly reversed the decline of mitochondrial OCR in LNCaP cells with enzalutamide treatment with androgen deprivation condition, while PPFIA4 knockdown aggravate the decline of OCR in C4-2B cells. We also characterized the effects of PPFIA4 on mitochondrial function by assessing the mitochondrial membrane potential, NADPH/ NADP^+^ ratio, and ROS production. Androgen deprivation led to the decrease of mitochondrial membrane potential (Fig. 4G) and NADPH/ NADP^+^ ratio (Fig. 4H) but the increase of mitochondrial ROS production in LNCaP cells (Fig. 4I), whereas PPFIA4 overexpression protected against the mitochondrial dysfunction caused by androgen deprivation (Fig. 4G-I). In contrast, mitochondrial membrane potential (Fig. 4J) and NADPH/ NADP^+^ ratio (Fig. 4K) decreased whereas the production of mitochondrial ROS increased in PPFIA4-depleted C4-2B cells (Fig. 4L). Furthermore, the addition of the ROS scavenging agent NAC can effectively reverse cell proliferation inhibition induced by PPFIA4 knockdown (Fig. 4M). Overall, PPFIA4 contributes to alleviation of mitochondrial dysfunction under androgen deprivation and enhances mitochondrial activity by balancing cellular redox homeostasis.

### PPFIA4 enhances the mitochondrial function via MTHFD2

One-carbon metabolism is one of the important pathways to maintain cellular redox balance. It has been reported that the increase of serine and one-carbon pathway metabolism can promote the occurrence of neuroendocrine prostate cancer (NEPC) [33]. Further transcriptome analyses on public datasets (GSE3325, GSE32269 and GSE6919) showed that several key genes of the one-carbon metabolism pathway, including MTHFD2, MTHFD1, SHMT2, and SHMT1 were upregulated in CRPC tissues compared with primary localized PCa (Supplementary Fig. S4). These results suggest that the level of one-carbon metabolism is altered in CRPC progression.

**Fig. 5:**
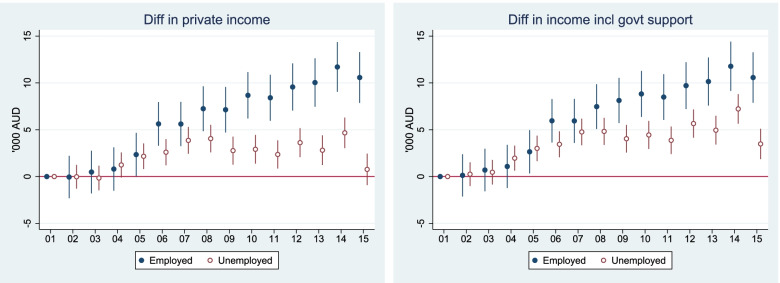
PPFIA4 interacts with MTHFD2 in mitochondria and accentuates mitochondrial function via MTHFD2.** A**. Identification of proteins that interact with PPFIA4 by Co-IP assays and mass spectrometry analysis. C4-2B cell lysates were immunoprecipitated with PPFIA4 antibody and subjected to SDS–PAGE. Coomassie blue staining were then performed, protein bands were cut and subjected to mass spectrometry. **B-C**. The binding potential between PPFIA4 and MTHFD2 in the whole cell lysates (Total, B), cytoplasmic fraction (Cyto) and mitochondrial fraction (Mito) were performed by Co-IP assays in C4-2B cells. IgG serves as negative control. COXIV and tubulin were used as mitochondrial and cytosolic markers. **D**. The binding potential between PPFIA4 and MTHFD2 was analyzed by Co-IP assays in LNCaP cells with or without androgen deprivation. IgG serves as negative control. **E**. The distribution of PPFIA4 (green) and MTHFD2 (red) was analyzed by immunofluorescence staining in LNCaP cells with or without androgen deprivation. Representative images are shown with a 10-μm scale bar. FBS, fatal bovine serum. CSS, charcoal-stripped serum. **F**. Measurement of OCR in C4-2B cells transfected with PPFIA4 overexpression plasmid for 24 hours and subsequent MTHFD2 siRNA for another 24 hours. Representative recordings of OCR during extracellular flow analysis (“Seahorse”) are shown in the up panel, and quantitative analysis of the calculated basal and maximum respiratory rates, ATP production rate and spare respiratory capacity are shown in the bottom panel. *p < 0.05, ***p < 0.001. m, minutes. **G-H**. Measurement of NADPH/NADP^+^ ratio (G) and ROS levels (H) in C4-2B cells transfected with PPFIA4 overexpression plasmid for 24 hours and subsequent MTHFD2 siRNA for another 24 hours. *p < 0.05, **p < 0.01. **I-K**. Cell counts (I), cell clone formation assays (J), and cell apoptosis assays (K) were performed in C4-2B cells co-transfected with PPFIA4 overexpression plasmid for 24 hours and subsequent MTHFD2 siRNA for another 24 hours. *p < 0.05, **p < 0.01, ***p < 0.001. d, days

To define the mechanism underlying the regulation of mitochondrial function by PPFIA4, we firstly analyzed the interactome of PPFIA4. Because MTHFD2 is the key enzyme for one-carbon metabolism in PCa progression [34], we selected MTHFD2 as the potential interactor with PPFIA4 in mitochondria (Fig. 5A). Co-IP assays showed that PPFIA4 physically interacts with MTHFD2 in C4-2B cells (Fig. 5B). Cytoplasmic and mitochondrial extracts were then fractionated from C4-2B cells and subjected to Co-IP assays. Of note, the PPFIA4-MTHFD2 complex is mainly localized in mitochondria (Fig. 5C), in which MTHFD2 exert its enzymatic activity [35]. Furthermore, Co-IP assays and immunofluorescent staining showed that the binding between PPFIA4 and MTHFD2 increased in response to ADT (Fig. 5D-E). These results suggest that PPFIA4 mainly acts to complex with MTHFD2 and affect MTHFD2 enzyme activity, especially under the androgen deprivation condition. As shown in Supplementary Fig. 5A, PCa patients with MTHFD2 overexpression were characterized by worse overall survival in the GEPIA prostate cohort. Furthermore, silencing MTHFD2 can effectively inhibit cell growth and clone formation of C4-2B cells (Supplementary Fig. S5B-D).

We then demonstrated that siRNA knockdown of MTHFD2 could attenuate the increased OCR (Fig. 5F) and NADPH/ NADP^+^ ratio (Fig. 5G) but restore the decreased ROS production (Fig. 5H) caused by PPFIA4 overexpression. Moreover, the increase of cell proliferation, colony formation and the decrease of cell apoptosis caused by PPFIA4 overexpression significantly decreased when MTHFD2 was silenced (Fig. 5I-K). Collectively, these data suggest that PPFIA4 can enhance mitochondrial function through MTHFD2 in PCa cells.

### PPFIA4 activates MTHFD2 via Src-mediated phosphorylation

**Fig. 6:**
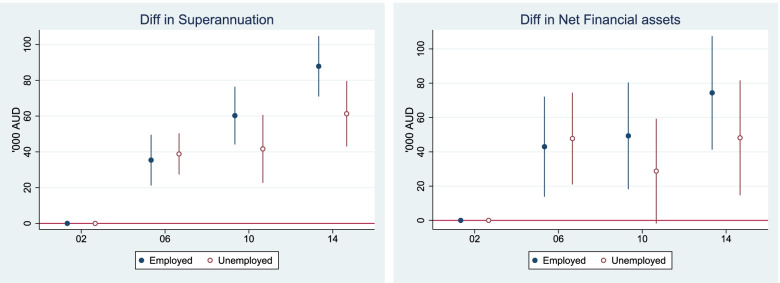
PPFIA4 activates MTHFD2 via Src-mediated phosphorylation. **A**. Cell lysates from LNCaP cells with or without androgen deprivation were subjected to immunoprecipitation using anti-phospho-serine/threonine/tyrosine specific antibodies, followed by immunoblotting with indicated antibodies. p-Ser, phospho-serine. p-Thr, phospho-threonine. p-Tyr, phospho-tyrosine. IgG serves as negative control. GAPDH was used as a loading control. **B**. Cell lysates from LNCaP cells (left) transfected with empty vector or PPFIA4 overexpression plasmid and C4-2B (right) cells transfected with siNC or siPPFIA4 were subjected to immunoprecipitation with anti-phospho-tyrosine antibody, followed by immunoblotting with indicated antibodies. IgG serves as negative control. GAPDH was used as a loading control. **C**. Cell lysates from HEK293T cells transfected with flag-MTHFD2/WT, flag- MTHFD2/Y84A, flag-MTHFD2/Y170A, flag-MTHFD2/Y234A, or flag- MTHFD2/Y304A plasmids were subjected to immunoprecipitation using anti-phospho-tyrosine antibody, followed by immunoblotting with indicated antibodies. **D-F**. Measurement of NADPH/NADP^+^ (D), ROS levels (E) and cell proliferation (F) in C4-2B cells transfected with siNC or siPPFIA4 for 24 hours and subsequent flag-MTHFD2/WT or flag-MTHFD2/Y170A for another 48 hours. **p < 0.01, *** *p* < 0.001. d, days. NS, no significance. **G**. Tyrosine phosphorylation levels of MTHFD2 were measured by immunoprecipitation and immunoblotting in C4-2B cells treated with Src inhibitor PP2 (10 μM) for 2 hours (top) or transfected with Src siRNA (siSrc) for 48 hours (bottom). IgG serves as negative control. GAPDH was used as a loading control. **H**. The binding potential between MTHFD2 and Src were performed in the total, cytosolic and mitochondrial lysates by Co-IP assays in C4-2B cells. IgG serves as negative control. COXIV and tubulin were used as mitochondrial and cytosolic markers. **I**. The binding potential between MTHFD2 and Src were performed by Co-IP assays in C4-2B cells transfected with siNC or siPPFIA4. IgG serves as negative control. GAPDH was used as a loading control. **J**. Tyrosine phosphorylation levels of MTHFD2 were measured by immunoprecipitation and immunoblotting in C4-2B cells transfected with empty vector or PPFIA4 overexpression plasmid and then treated with PP2 (top) or transfected with siNC or siSrc (bottom). IgG serves as negative control. GAPDH was used as a loading control. **K**. A putative schematic diagram illustrating the role of PPFIA4 in contributing to CRPC. PPFIA4 proteins translocate mitochondria in response to androgen deprivation, activates MTHFD2 via Src-mediated phosphorylation, and consequently promotes the production of NADPH and reduces the excessive ROS generation, which could enhance mitochondrial activity and promote CRPC progression

We next investigated the effects of PPFIA4 on MTHFD2 in PCa cells. As the expression of MTHFD2 at both mRNA and protein levels failed to respond to siRNA knockdown or ectopic overexpression of PPFIA4 (Supplementary Fig. S6A-B), PPFIA4 might regulate MTHFD2 expression at post-translational levels. It has been reported that SIRT3 can deacetylate MTHFD2 and promote its enzymatic activity in colorectal cancer cells [36]. However, no observable change of acetylated MTHFD2 was detected after overexpression or depletion of PPFIA4 in PCa cells (Supplementary Fig. S6C). Notably, we observed that the level of tyrosine phosphorylation of MTHFD2 increased significantly when LNCaP cells were challenged by ADT (Fig. 6A). More specifically, the levels of tyrosine phosphorylated MTHFD2 increased pronouncedly by PPFIA4 overexpression (Fig. 6B left) but decreased by PPFIA4 depletion (Fig. 6B right). Amino acid sequence analysis revealed the presence of four putative tyrosine residues in MTHFD2, which includes Y84, Y170, Y234, and Y304. Next, we made the tyrosine to alanine substitutions at those sites (Y84A, Y170A, Y234A, and Y304A) to disrupt phosphorylation at the tyrosine residues. Subsequent western blotting analysis revealed that there was a significant decrease in tyrosine phosphorylation of MTHFD2 protein in MTHFD2-Y170A transfected cells compared to the wild-type MTHFD2 transfection (Fig. 6C). Furthermore, MTHFD2-WT, but not MTHFD2-Y170A, could rescue the mitochondrial dysfunction (Fig. 6D-E) and decreased cell growth (Fig. 6F) caused by silencing PPFIA4 in C4-2B cells. These results suggest that the phosphorylation of mitochondrial MTHFD2 at Tyr170 pinpoints the regulatory role of PPFIA4 for its enzymatic activity and mitochondrial function.

To elucidate the mechanism by which PPFIA4 regulates MTHFD2 tyrosine phosphorylation, we identified MTHFD2 as the substrate of Src by PhosphoMotif Finder (http://hprd.org/PhosphoMotif_finder/). Intra-mitochondrial Src is observed in various tumor cells, and several mitochondrial proteins had been identified as substrates for Src kinases [37, 38]. Therefore, we proposed that PPFIA4 might promote tyrosine phosphorylation of MTHFD2 through Src. The inhibition of Src by its inhibitor (PP2) or specific siRNA (Supplementary Fig. 6D) decreased the levels of tyrosine-phosphorylated MTHFD2 in C4-2B cells (Fig. 6G). Moreover, the results showed that MTHFD2 co-immunoprecipitated with Src and the complex was mainly located in mitochondria (Fig. 6H). In addition, Co-IP assays showed that the interaction between MTHFD2 and Src was reduced by PPFIA4 depletion in C4-2B cells but enhanced by PPFIA4 overexpression in LNCaP cells (Fig. 6I; Supplementary Fig. S6E). Furthermore, the increased levels of MTHFD2 tyrosine phosphorylation resulting from PPFIA4 overexpression could be attenuated by Src inhibition in C4-2B cells (Fig. 6J). Based on these data, we suggest that PPFIA4 activates MTHFD2 via Src-mediated phosphorylation to enhances mitochondrial activity, promotes cell proliferation, and accelerates castration resistance of PCa (Fig. 6K).

### MTHFD2 inhibitor is effective in delaying CRPC growth both *in vitro* and *in vivo*

**Fig. 7:**
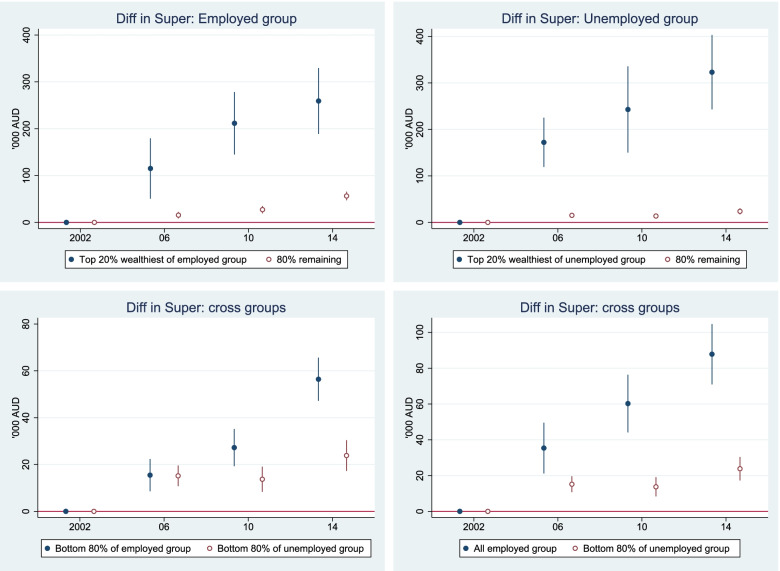
DS18561882 inhibits CRPC progression *in vitro* and *in vivo.* **A-B**. Cell proliferation and cell apoptosis assays of LNCaP cells transfected with empty vector or PPFIA4 overexpression plasmid (A) and C4-2B cells transfected with siNC or siPPFIA4 (B) with or without DS18561882 (50μM) treatment. **p < 0.01, ***p < 0.001. d, days. **C-E**. Castrated mice possessing xenografts (LNCaP-Vector and LNCaP-PPFIA4) received vehicle control or DS18561882 treatment (100 mg/kg, *n =* 5/group, p.o.). Caliper measurements were taken twice every week to obtain tumor volume (C). Tumors were collected and weighed (D) after the mice were sacrificed. IHC staining of Ki67 (E) on tumor slide from each group is shown. **p < 0.01, ***p < 0.001. Scale bars, 20 μm. p.o., peros. **F-G**. Cell proliferation (F) and cell apoptosis assays (G) of C4-2B (left) and LNCaP cells (right) treated with DMSO, enzalutamide (10 μM), DS18561882 (50 μM) alone or in combination. *p < 0.05, **p < 0.01, ***p < 0.001, ****p < 0.0001. d, days. ENZ, enzalutamide. COM, combination. **H-J**. Castrated mice bearing xenografts (C4-2B cells) were treated with vehicle control, enzalutamide (10 mg/kg, p.o.), DS18561882 (100 mg/kg, p.o.), or in combination (*n =* 5/group). Tumor volume was measured (H) and then weighed (I) when mice were sacrificed. IHC staining of Ki67 (J) on tumor slides from each group is shown. ***p < 0.001, ****p < 0.0001. Scale bars, 20 μm. p.o., peros. ENZ, enzalutamide. COM, combination

Considering the important role of PPFIA4, we then evaluated whether PPFIA4 promoted PCa progression in a MTHFD2-dependent manner. DS18561882, a highly potent isozyme-selective inhibitor of MTHFD2 showed strong cell-based activity and a good oral pharmacokinetic profile [39]. As shown in Fig. 7A, DS18561882 could inhibit cell proliferation but increase cell apoptotic rates even in the presence of PPFIA4 overexpression in LNCaP cells. Furthermore, DS18561882 treatment and PPFIA4 knockdown in C4-2B cells additively inhibited cell proliferation but increased cell apoptic rates (Fig. 7B; Supplementary Fig. S7A). We next evaluated the effects of DS18561882 on tumor growth *in vivo*. When castrated mice carrying LNCaP or VCaP xenografts (vector and PPFIA4) were treated with DS18561882, the inducible effects of PPFIA4 on tumor growth could hardly be detected, as evidenced by tumor growth (Fig. 7C; Supplementary Fig. S7B), tumor volume (Fig. 7D; Supplementary Fig. S7C), and the proliferation index Ki67 (Fig. 7E; Supplementary Fig. S7D). In summary, DS18561882 can significantly inhibit CRPC progression caused by the high expression of PPFIA4. Of note, no significant change in body weight and morphological abnormalities were identified in important organs, such as the liver and kidney (Supplementary Fig. S7E-F).

Given the increased PPFIA4 expression in response to androgen deprivation in PCa cells and its role to promote CRPC progression, we hypothesized that blocking of PPFIA4-MTHFD2 axis in combination with new first-line endocrine therapy could be ideal to prevent CRPC progression. As shown in Fig. 7F-G, DS18561882 synergized with enzalutamide to inhibit cell proliferation and induce a strong cell apoptotic response. More importantly, tumor growth inhibition was more significant in the combination treatment of DS18561882 and enzalutamide when compared with single treatment strategy *in vivo* (Fig. 7H-J). These data suggested that DS18561882 and enzalutamide worked in synergy to delay CRPC progression both *in vitro* and *in vivo*.

## Discussion

Mitochondria are critical organelles for energy production allowing cancer cells to survive, proliferate and disseminate under an altered microenvironment [40]. Grupp *et al.* [11] found that higher numbers of mitochondria were associated with increasing tumor grade and stage, suggesting that increased mitochondrial content is necessary or supportive for prostate cancer development and progression [41]. ADT is known to initially suppress tumor growth but eventually fails as the ADT resistant tumors recur and develop with time, however, little is known about the changes of mitochondria during ADT. It has been demonstrated that testosterone deprivation accelerated cardiac mitochondrial dysfunction [42] and mitochondrial membrane was impaired when PCa cells were challenged by androgen deprivation [12, 13]. Mitochondria are also important sites for ROS production [43]. Specifically, androgen deprivation in PCa cells can increase ROS production [44], indicating the occurrence of mitochondrial dysfunction**.** Meanwhile, CRPC progression is accompanied by elevated levels of some antioxidant proteins, such as TRX1 and TXNDC9 [45, 46], indicating CRPC requires enhanced protective adaptations to buffer against excessive ROS elevation in response to androgen deprivation. As mitochondria possess mechanisms to clear or utilize waste products, mitochondria-mediated oxidative stress may play an important role. Therefore, identifying the pathological mechanisms that could protect mitochondrial function and enhance its activity under androgen-deprived condition may provide a novel therapeutic target for PCa.

Of liprin-α family members, liprin-α1 has been shown to promote cell spreading by affecting integrins [47, 48], whereas the other liprin-α family members remain poorly characterized. It is reported that liprin-α4 is up-regulated under hypoxic conditions and is directly regulated by HIF-1α in renal cell carcinoma [18]. Moreover, As the intracellular effectors of receptor protein tyrosine phosphatase-leukocyte antigen related receptor F (LAR-RPTP), PPFIA4 interacts with the catalytically inactive D2 domain of LAR-RPTP via their highly conserved C-terminal three sterile alpha motif domains and promotes its phosphatase activity [49]. There is emerging evidence indicating that PPFIA4 plays an important role in several malignancies including refractory pancreatic cancer [20], small cell lung cancer [21], and clear cell renal cell cancer [18]. In this study, we clearly demonstrated that PPFIA4 could promote CRPC progression by enhancing the mitochondrial function of PCa cells and served as a novel regulator for mitochondrial function in CRPC progression.

PCa has a unique metabolism signature that unlike most solid tumors, it is not highly glycolytic (at least at early stages) [33]. PCa progression, such as neuroendocrine differentiation, is characterized by metabolic reprograming that offers competitive advantages through regulating the one-carbon pathway [33]. The central role of the one-carbon pathway in cell growth and transformation is due to its pleiotropic function in possession of key metabolic pathways, including the synthesis of nucleotides needed for cell growth, the biosynthesis of S-adenosyl methionine for promoting epigenetic changes conducive to neuroendocrine differentiation, and the production of NAD(P) H and glutathione for maintaining an adequate redox balance in cancer cells [50]. MTHFD2, a key enzyme for one-carbon metabolism, is essential for protecting mitochondrial function. Our studies identified that MTHFD2 was an important binding partner of PPFIA4. Furthermore, the increase of antioxidant activity and cell proliferation induced by PPFIA4 overexpression can be largely reduced by silencing MTHFD2, which suggests that PPFIA4 meditates the mitochondrial ROS homeostasis and cell proliferation by enhancing mitochondrial activity through MTHFD2.

In addition to the identification of MTHFD2 as a key binding partner of PPFIA4, our studies showed that PPFIA4 expression, mitochondrion localization, and its complex formation with MTHFD2 were enhanced when LNCaP cells were challenged by androgen deprivation. Thus, the PPFIA4-MTHFD2 interaction can sense the androgen environment alteration and links androgen levels to mitochondrial metabolism and cell proliferation in PCa cells. Given the prominent roles of mitochondrial metabolism in regulating energy production and redox balance, the androgen deprivation responsive interaction between PPFIA4 and MTHFD2 likely serves as a key regulator in mitochondrial homeostasis and cellular proliferation of PCa *in vitro*.

The fact that mitochondria participate closely in PCa development makes them promising targets for anticancer therapy. It has been reported that novel mitochondria-targeted therapeutic agents, like Pentamidine, Atpenin A5, and Urupocidin c could efficiently suppress PCa progression [51–53]. Moreover, PAWI-2 can activate mitochondrial-controlled p53-dependent apoptotic signaling and possesses promising therapeutic potency in low-dose combination therapy with enzalutamide [54, 55]. In this study, we identified the PPFIA4-MTHFD2 axis as an important pathway to activate mitochondria metabolism and blocking this pathway by a specific inhibitor DS18561882 towards MTHFD2 could efficiently delay CRPC progression.

## Conclusion

In summary, our results demonstrate that PPFIA4 has the potential to promote CRPC progression by enhancing mitochondrial metabolism via MTHFD2. The co-treatment of DS18561882 and enzalutamide may achieve a better intervention effect for suppressing CRPC progression. These findings advance our understanding of CRPC development and provide an attractive approach for intervening CRPC progression.

## Supplementary Information

**Additional file 1.** Supplemental Materials and Methods.

**Additional file 2: Table S1.** Primers used in this study. **Table S2.** Antibodies used in this study. **Table S3.** siRNAs used in this study.

**Additional file 3: **Supplementary Figures and Legends. **Figure S1.** PPFIA4 does not alter expression of AR and its target genes in PCa cells. **Figure S2.** PPFIA4 promotes PCa cell growth *in vitro*. **Figure S3.** Androgen deprivation induces mitochondrial dysfunction. **Figure S4.** The increased expression of several key genes involving one-carbon metabolism in CRPC. **Figure S5.** MTHFD2 overexpression promotes CRPC cell proliferation and is associated with poor prognosis in PCa patients. **Figure S6.** PPFIA4 exerts no significant effect on MTHFD2 expression. **Figure S7.** DS18561882 significantly suppresses PCa cell growth *in vitro* and *in vivo*.

## Data Availability

All data and material during the current study are available from the corresponding author on reasonable request.

## Abbreviations

CRPC: Castration-resistant prostate cancer
PPFIA4: Protein tyrosine phosphatase receptor type F polypeptide interacting protein alpha 4
MTHFD2: Methylene- tetrahydrofolate dehydrogenase 2
ADT: Androgen deprivation therapy
PCa: Prostate cancer
AR: Androgen receptor
ROS: Reactive oxygen species
CSS: Charcoal-stripped serum
OCR: Oxygen consumption rate
NAC: N-acetyl-L-cysteine
