# Supplementary material for: PPFIA4 promotes castration-resistant prostate cancer by enhancing mitochondrial metabolism through MTHFD2
Source: J Exp Clin Cancer Res. 2022 Apr 5;41:125. doi: 10.1186/s13046-022-02331-3 (PMC8985307; doi:10.1186/s13046-022-02331-3)
Supplement: Supplementary file 1 — Additional file 1. Supplemental Materials and Methods. [file 13046_2022_2331_MOESM1_ESM.docx]

**Supplemental Materials and Methods**

**Cell proliferation, colony formation and flow cytometry analysis**

To determine the effects of PPFIA4 on cell proliferation of PCa cells, cell counts were carried out in triplicate using a hemocytometer. Cellular proliferation was also measured by Cell-Light™ EdU DNA Cell Proliferation (EdU) assays (Ribobio, Guangzhou, China) and clonal formation assays. Cell apoptosis was determined by flow cytometry following the manufacturer’s protocol (BB-4101, BestBio) and was analyzed on a BD FACS Calibur Cytometer and the included software.

**PSA ELISA**

assay was carried out using culture supernatant 72 hours after

siRNA/miRNA duplex transfection, using Human PSA ELISA

Kit (Abazyme), according to the manufacturer's instructions

and normalized to the MTT assay

assay was carried out using culture supernatant 72 hours after

siRNA/miRNA duplex transfection, using Human PSA ELISA

Kit (Abazyme), according to the manufacturer's instructions

and normalized to the MTT assay

assay was carried out using culture supernatant 72 hours after

siRNA/miRNA duplex transfection, using Human PSA ELISA

Kit (Abazyme), according to the manufacturer's instructions

and normalized to the MTT assay

LNCaP cells were treated with R1881 or androgen deprivation. The PSA level in the culture supernatants was collected and quantified by ELISA assay (Anogen, Mississauga, Ontario, Canada) based on the manufacturer’s instructions.

**Immunofluorescence (IF)**

Proteins were detected using goat anti-mouse Alexa Fluor 647 and goat anti-rabbit Alexa Fluor 488 (P0176, P0191, Beyotime Biotechnology, Jiangsu, China). Mitochondria were stained using MitoTracker Deep Red FM (40743ES50, Yeasen). Images were processed under a confocal microscope (FV3000, OLYMPUS, Tokyo, Japan).

**Chromatin immunoprecipitation (ChIP)**

ChIP was carried out using the ChIP assay kit (Beyotime Biotechnology, Jiangsu, China) following manufacturer’s instructions. Briefly, Chromatin from pretreated cells was fixed with 1% formaldehyde for 10 min at room temperature. DNA was broken into 200–1000 bp fragments using a sonicator (10^6^ cells in 250 μL volume; ultrasound 15s, stop 15s, repeated eight times). Chromatin was immunoprecipitated with anti-AR (Abcam, Cambridge, MA, USA) and control anti-IgG (St. Louis, MO, USA) antibodies. The association of AR with PPFIA4 was measured by qRT-PCR (pre-denaturation at 95 °C for 5 min; followed by 95 °C for 30 s, 65 °C for 30 s, and 72 °C for 30 s, 35 cycles) using immunoprecipitated chromatin from cells with the indicated primers listed in **Supplementary Table 1**.

**Luciferase reporter gene assay**

Cells were seeded in triplicate in 24-well plates and co-transfected with the Renilla expression plasmid pRL-TK and the reporter constructs for PPFIA4 wile type or PPFIA4 mutant promoters. Luciferase activities were measured using Dual-Luciferase Reporter Assay System (Promega, Madison, WI, USA). Luciferase activity was normalized to the luciferase activity of Renilla expression in the cells.

**Mitochondrial morphology analysis**

LNCaP cells (1 × 10^6^ cells) were cultured under androgen deprivation or not and transfected PPFIA4 overexpression plasmid for 48 hours. Then cells were washed, harvested, and fixed at 4°C for 24 hours with Fixing Solution (G1102, Servicebio). The cells were then post-fixed in 1% osmium tetroxide, dehydrated in a graded series of ethanol, infiltrated, and embedded in EMBed. Ultrathin sections were evaluated using a HT7700 transmission electron microscope (HITACHI).

**Mitochondrial membrane potential (MMP)**

MMP was measured using the dual fluorescence dye JC-1 (5,5′,6,6′-tetrachloro-1,1′,3,3′-tetraethylbenzimidazolyl-carbocyanine iodide) (C2006, Beyotime Biotechnology, Jiangsu, China). The fluorescence intensities of JC-1 monomers (Ex.490 nm, Em.530 nm) and aggregates (Ex.525 nm, Em.590 nm) were measured using a SpectraMax iD3 Multi-Mode Microplate Reader (Molecular Devices, US). MMP was measured as the ratio of JC-1 aggregate to monomers.

**Cellular ROS assay and NADPH/NADP levels assay**

Intracellular ROS production was evaluated using 2’ ,7’ -dichlorofluorescein diacetate (DCFH-DA; BestBio, BB-4705-2). Cells were incubated with DCFH-DA at 37 °C for 20 min to load the fluorescent dye and then washed with serum-free medium three times to measure the fluorescence (Ex.488 nm, Em.525 nm) by the SpectraMax iD3 Multi-Mode Microplate Reader (Molecular Devices, US). The intracellular levels of NADPH and total NADP were determined by using the NADP/NADPH-Glo Kit (Promega, Madison, WI, USA) according to the manufacturer's instructions.
