# Supplementary material for: PPFIA4 promotes castration-resistant prostate cancer by enhancing mitochondrial metabolism through MTHFD2
Source: J Exp Clin Cancer Res. 2022 Apr 5;41:125. doi: 10.1186/s13046-022-02331-3 (PMC8985307; doi:10.1186/s13046-022-02331-3)
Supplement: Supplementary file 2 — Additional file 2: Table S1. Primers used in this study. Table S2. Antibodies used in this study. Table S3. siRNAs used in this study. [file 13046_2022_2331_MOESM2_ESM.docx]

| **Supplementary Table S1. Primers used in this study.** | |  |
| --- | --- | --- |
| **Gene** | **Forward** | **Reverse** |
| PPFIA4 | GAAACACCAGCTGCTTGAA | TGATGGCACCACTCTTGAC |
| MTHFD2 | GTTGGTTGGAGATGTGGAT | AGACTTCAGCACTTCTCG |
| PSA | GCCTGGATCTGAGAGAGATATCATC | ACACCTTTTTTTTTCTGGATTGTTG |
| KLK2 | CCATGCCTGGAGACATATCA | TCCAGCACATGTCACTCTCC |
| TMPRSS2 | GGTAAACTCTCCCTGCCACA | TACTCCAGGAAGTGGGGATG |
| CKS2 | CACTACGAGTACCGGCATGTT | CACCAAGTCTCCTCCACTCC |
| LPL | AGTAGCAGAGTCCGTGGCTA | ATTCCTGTTACCGTCCAGCC |
| SMC4 | ATGGGCGAAAAAGATGACCG | ACTTTGCTTCCACCACCAGT |
| **ChIP** |  |  |
| PSA | GCCTGGATCTGAGAGAGATATCATC | ACACCTTTTTTTTTCTGGATTGTTG |
| P1 | GTGAGCCTGGGTCAGTCAAA | TTCTCCCCTCCTGCCTCAAA |
| P2 | AGAGAAGGTTGTATGTGTCACTG | TGCAATGTGTTCATGTGTGTGTA |
| P3 | CAGGAGAGCAAGCGTGAAAG | ACTCGCCCTTGTTCCTAGT |

| **Supplementary Table S2. Antibodies used in this study.** | |
| --- | --- |
| **Antibody** | **Cat no.** |
| GAPDH | cat no. ab181602; Abcam |
| COXIV | cat no.sc-376731; Snata |
| PPFIA4 | cat no. ab188147; Abcom |
| MTHFD2 | cat no.12270-1-AP; Proteintech |
| SRC | cat no.11097-1-AP; Proteintech |
| Flag | cat no. ab205606; Abcam |
| β-Tublin | cat no.10094-1-AP; Proteintech |
| AR | cat no. 5153; Cell Signaling |
| Phosphotyrosine (pY) | cat no.clone 4G-10 Platinum; Upstate, Millipore |

| **Supplementary Table S3. siRNAs used in this study.** | |
| --- | --- |
| **siRNA** | **Sequence** |
| siPPFIA4 #1 | AGGAGATGGTGTCATTGACCAG |
| siPPFIA4 #2 | GAGAUUGAGACGCGUGUAA |
| siAR | AAGAAGGCCAGUUGUAUGGAC |
| siMTHFD2#1 | GGAUGCUUCACUUUGUCAA |
| siMTHFD2#2 | GCAGTTGAAGAAACATACAAT |
| siSRC#1 | GGUUCACCAUCAAGUCAGA |
| siSRC#2 | GCCUCUCAGUGUCUGACUUTT |
