# Supplementary material for: PPFIA4 promotes castration-resistant prostate cancer by enhancing mitochondrial metabolism through MTHFD2
Source: J Exp Clin Cancer Res. 2022 Apr 5;41:125. doi: 10.1186/s13046-022-02331-3 (PMC8985307; doi:10.1186/s13046-022-02331-3)
Supplement: Supplementary file 3 — Additional file 3: Supplementary Figures and Legends. Figure S1. PPFIA4 does not alter expression of AR and its target genes in PCa cells. Figure S2. PPFIA4 promotes PCa cell growth in vitro. Figure S3. Androgen deprivation induces mitochondrial dysfunction. Figure S4. The increased expression of several key genes involving one-carbon metabolism in CRPC. Figure S5. MTHFD2 overexpression promotes CRPC cell proliferation and is associated with poor prognosis in PCa patients. Figure S6. PPFIA4 exerts no significant effect on MTHFD2 expression. Figure S7. DS18561882 significantly suppresses PCa cell growth in vitro and in vivo. [file 13046_2022_2331_MOESM3_ESM.docx]

**Supplementary Figures and Legends**

**
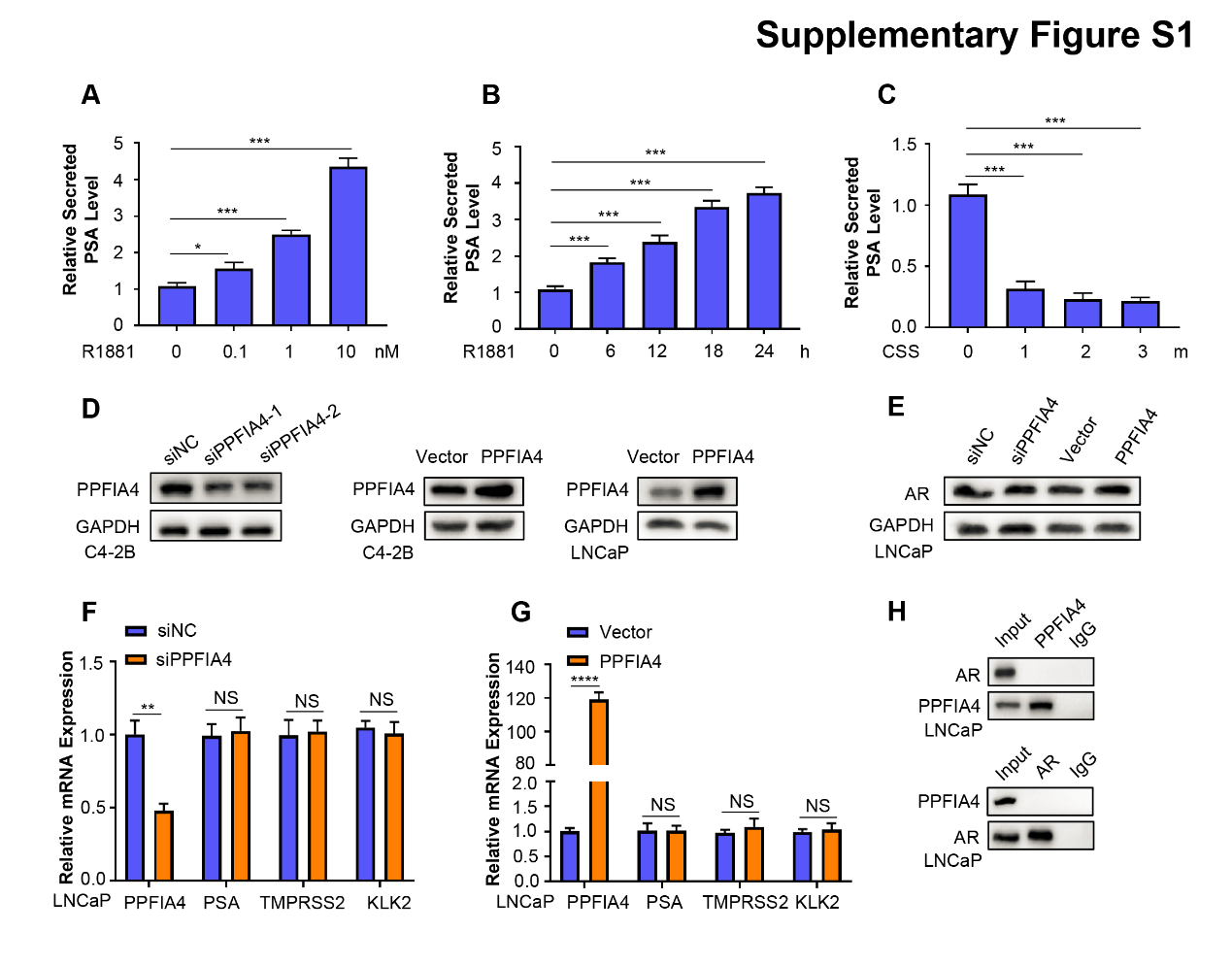
**

**Supplementary Figure S1. PPFIA4 does not alter expression of AR and its target genes in PCa cells.**

A-B. PSA levels measured in the culture medium of LNCaP cells with androgen treatment by ELISA. LNCaP cells were treated with 0.1, 1, and 10 nM R1881 for 24 hours (A) or treated with 1 nM R1881 at the indicated time points (B). Secreted PSA levels relative to untreated controls are indicated. **p* < 0.05, ****p* < 0.001 based on the Student’s *t*-test. h, hours.

C. PSA levels measured in the culture medium of LNCaP cells with prolonged androgen-deprivation (1, 2, and 3 months) by ELISA. Secreted PSA levels relative to untreated controls are indicated. ****p* < 0.001 based on the Student’s *t*-test. m, months. CSS, charcoal-stripped serum.

D. The protein levels of PPFIA4 were evaluated in PCa cells with PPFIA4 knockdown or overexpression. Total protein was extracted and evaluated by western blotting 48 hours after transfection. GAPDH was used as a loading control.

E-G. The protein levels of AR (E) and the mRNA levels of AR target genes (PSA, TMPRSS2, and KLK2) (F-G) were determined by western blotting and qRT-PCR in LNCaP cells with PPFIA4 knockdown or overexpression. GAPDH was used as a loading control. ****p* < 0.001, *****p* < 0.0001.

H. Co-immunoprecipitation (Co-IP) assays were performed to evaluate the binding potential between PPFIA4 and AR in LNCaP cells. IgG serves as negative control.


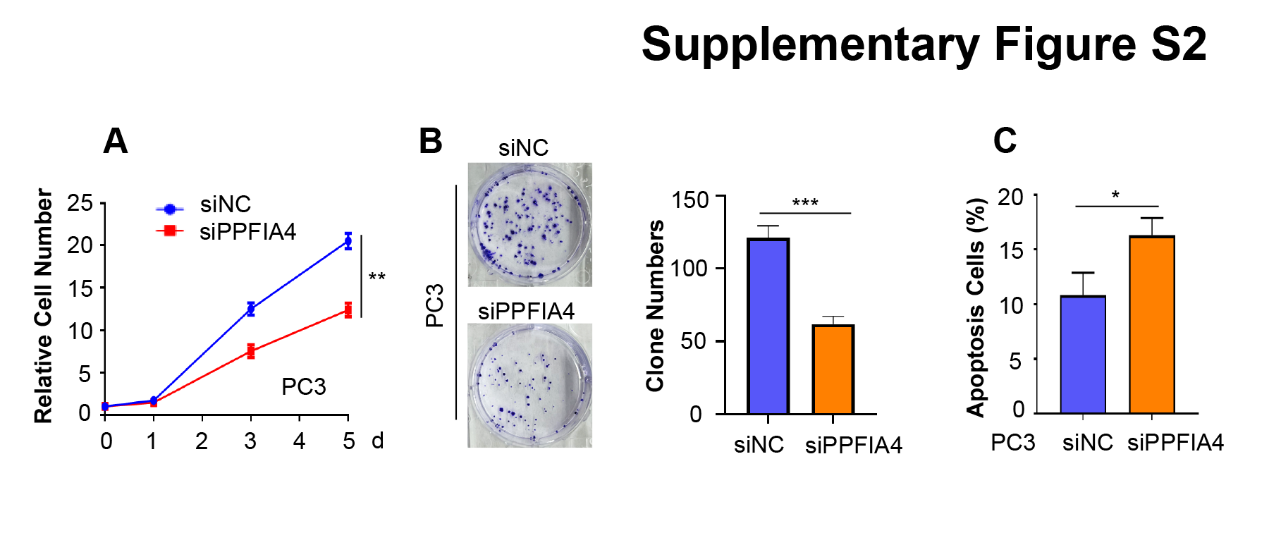


**Supplementary Figure S2. PPFIA4 promotes PCa cell growth *in vitro*.**

1. Cell proliferation was assessed by cell counts at indicated time points in PC3 cells transfected with negative control (siNC) or PPFIA4 siRNA (siPPFIA4). Cell number was counted at the indicated time points and all the numbers were normalized to day 0. Data shown are presented as means ± SEM of triplicate wells and are representative of at least three replicate experiments. ***p* < 0.01 based on the Student’s *t*-test. d, days.

B-C. Colony formation assays (B) and cell apoptosis assays (C) were performed in PC3 cells with PPFIA4 knockdown. Representative images are shown in the left panel and quantitative analysis is shown in the right panel. * *p* < 0.05. ***p* < 0.01, ****p* < 0.001.


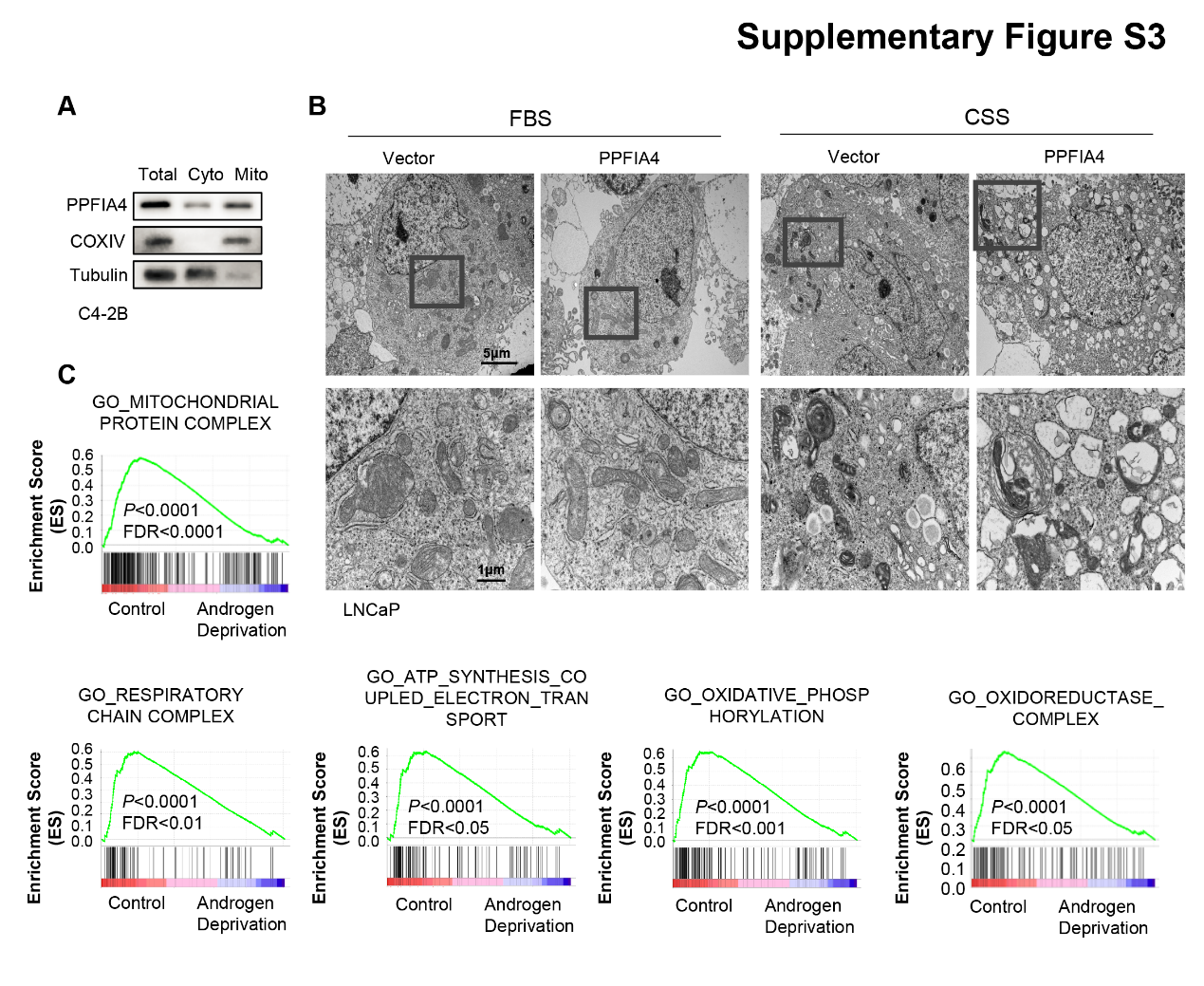


**Supplementary Figure S3. Androgen deprivation induces mitochondrial dysfunction.**

A. The protein levels of PPFIA4 in total cell lysates (Total), cytosolic fraction (Cyto), and mitochondrial fraction (Mito) were analyzed by western blotting in C4-2B cells. COXIV and tubulin were used as mitochondrial and cytosolic markers.

B. Representative electron microscopy images of mitochondrial morphology in PPFIA4 overexpressed LNCaP cells with or without androgen deprivation. Scale bars, 5 μm. Magnified images from the regions marked by rectangles in the top panel was showed in the bottom panel. Scale bars, 1 μm.

C. Gene Set Enrichment Analysis (GSEA) enrichment plot of the mitochondrial function-related gene sets. LNCaP cells were cultured in an androgen-deprived medium for 3 months. We collected the total RNA and preformed microarray analyses. GSEA analysis was then carried out to examine the enrichment of the mitochondrial function-related gene sets. ES, enrichment score. FDR, false discovery rate.


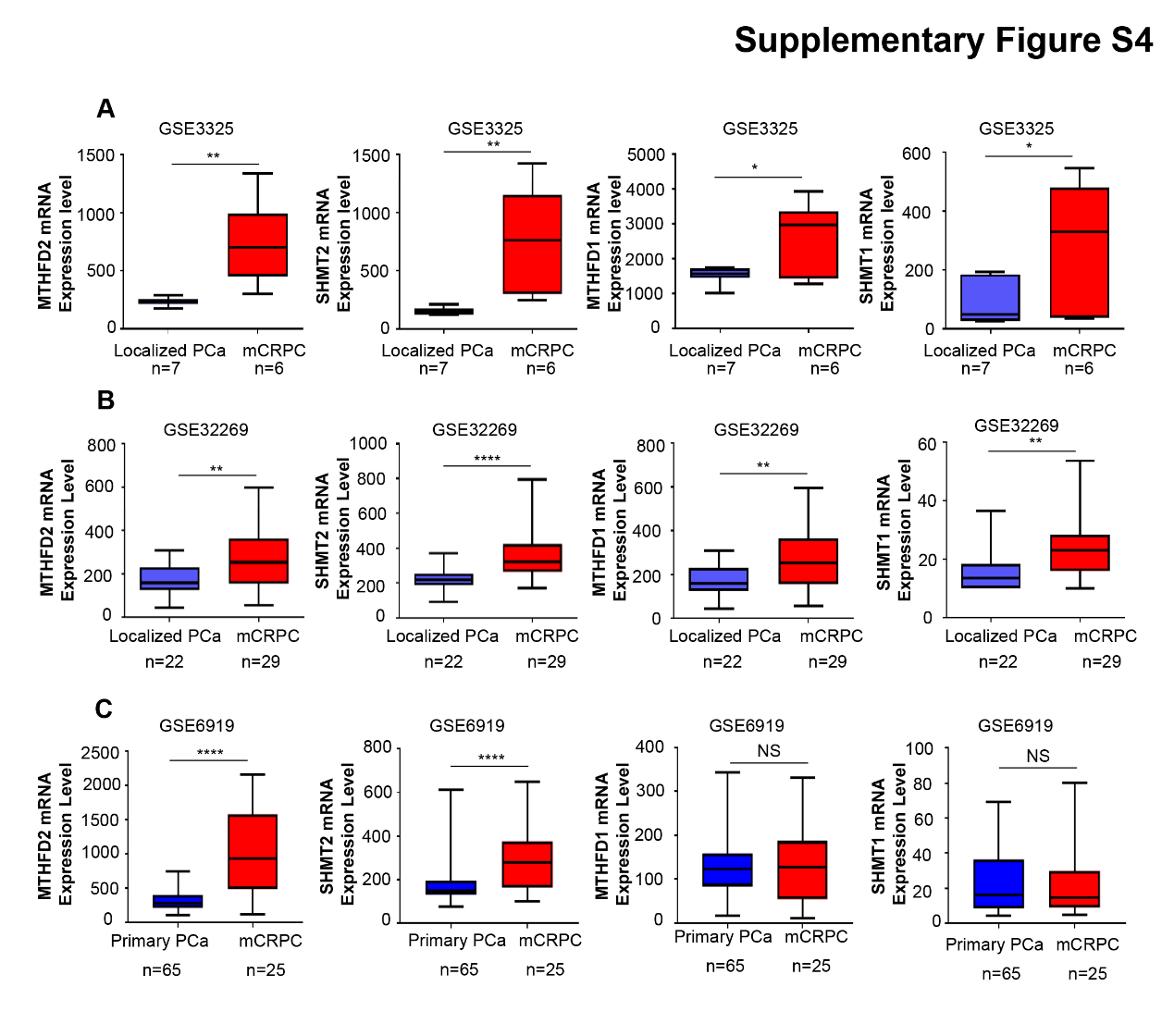


**Supplementary Figure S4. The increased expression of several key genes involving one-carbon metabolism in CRPC.**

A-C. Expression of MTHFD2, MTHFD1, SHMT2 and SHMT1 in metastatic CRPC (mCRPC) tissues compared with primary localized PCa samples in GSE3325, GSE32269, and GSE6919 public datasets. ** p* < 0.05, ***p* < 0.01, *****p* < 0.0001. NS, no significance.


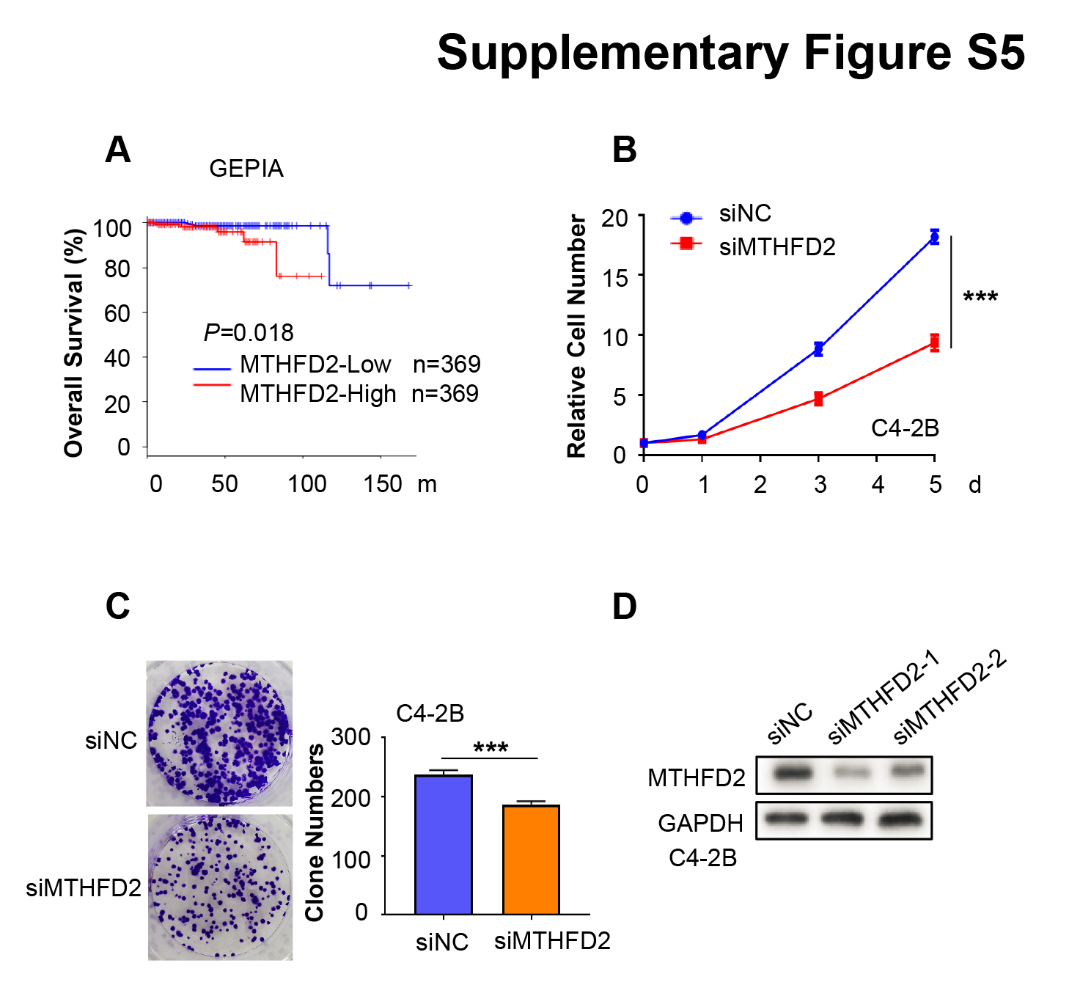


**Supplementary Figure S5. MTHFD2 overexpression promotes CRPC cell proliferation and is associated with poor prognosis in PCa patients.** A. Kaplan–Meier survival analysis of PCa cases from GEPIA prostate cohort according to high and low MTHFD2 expression. We arbitrarily defined the top 50% as the MTHFD2 high group and the bottom 50% as the MTHFD2 low group. m, months.

B-C. Cell proliferation was determined by cell counts (B) and colony formation assays (C) in C4-2B cells transfected with siNC and MTHFD2 siRNA (siMTHFD2). ****p* < 0.001. d, days.

D. The protein levels of MTHFD2 were analyzed in C4-2B cells transfected with siNC or siMTHFD2. Total protein was extracted and evaluated by western blotting 48 hours after transfection. GAPDH was used as a loading control.

**
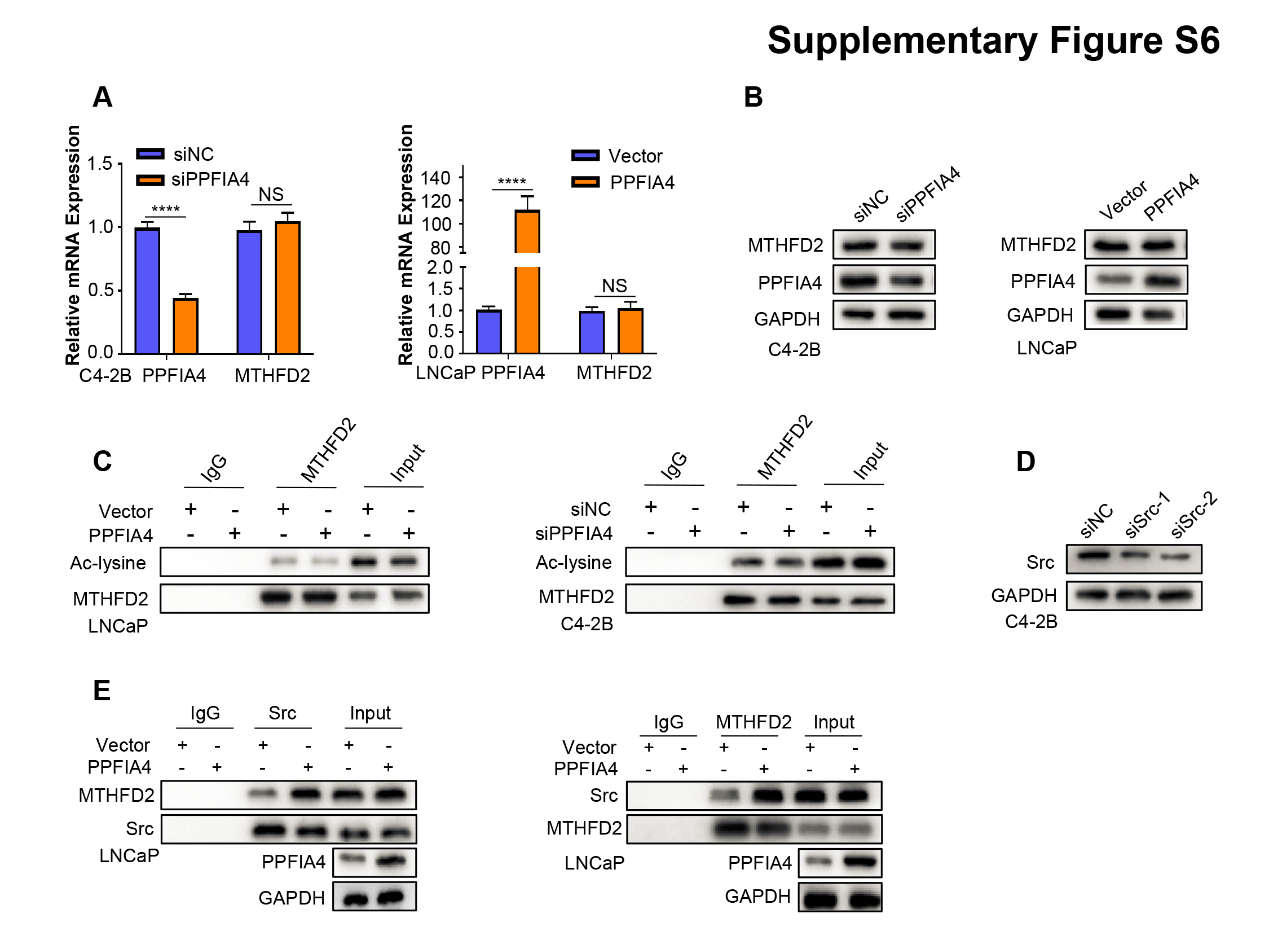
**

**Supplementary Figure S6. PPFIA4 exerts no significant effect on MTHFD2 expression.**

A-B. The mRNA and protein levels of MTHFD2 in PCa cells with PPFIA4 knockdown or overexpression were analyzed by qRT-PCR (A) and western blotting (B). GAPDH was used as a loading control. *****p* < 0.0001. NS, no significance.

C. The acetylation levels of MTHFD2 were detected by immunoprecipitation and western blotting in C4-2B cells with PPFIA4 overexpression or knockdown. IgG serves as negative control.

D. The protein levels of Src were analyzed in C4-2B cells transfected with siNC or Src siRNA (siSrc). Total protein was extracted and evaluated by western blotting 48 hours after transfection. GAPDH was used as a loading control.

E. LNCaP cells were transfected with empty vector or PPFIA4 overexpression plasmid. Co-IP assays were then performed to evaluate the binding potential between MTHFD2 and Src. GAPDH was used as a loading control. IgG serves as negative control.


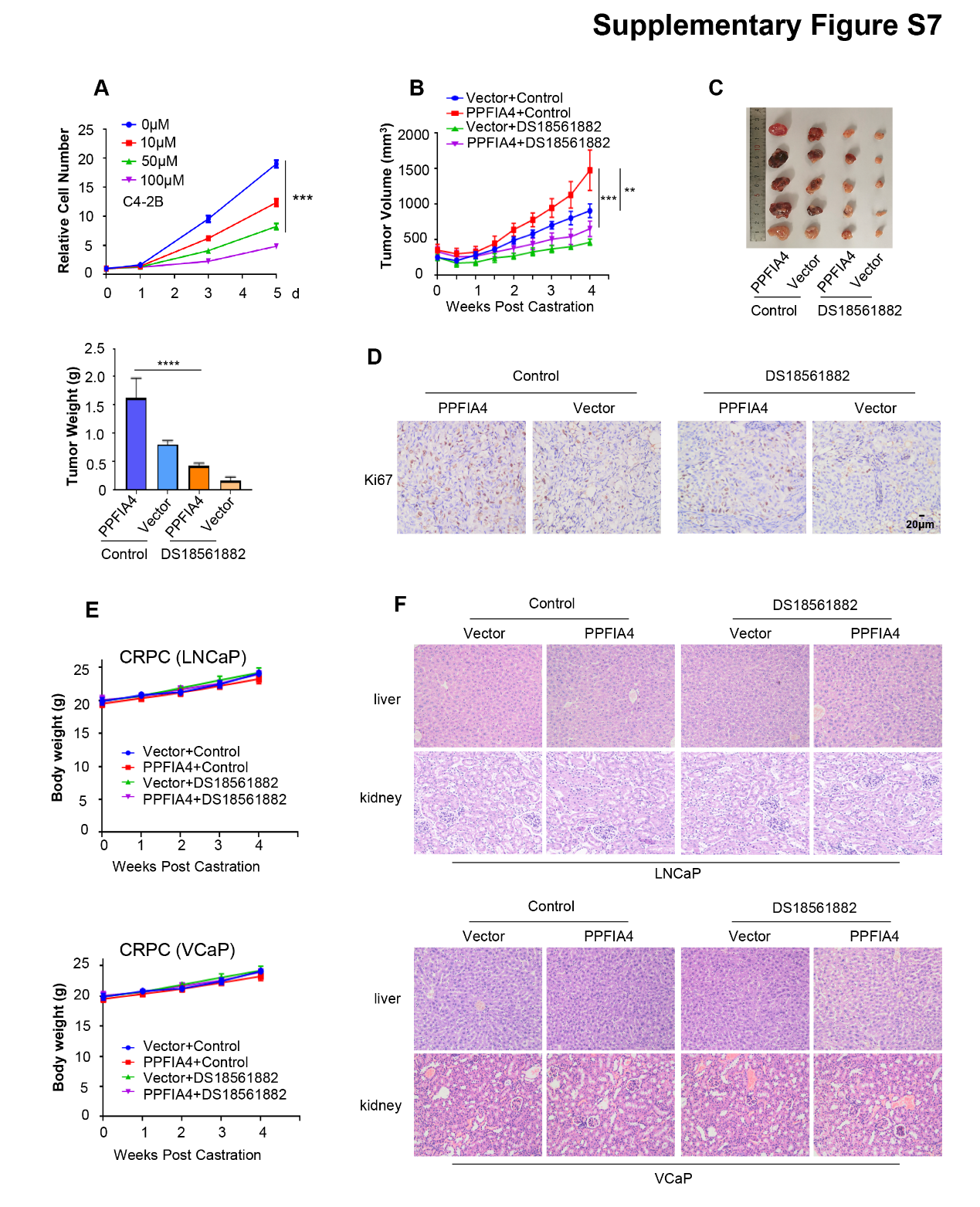


**Supplementary Figure S7. DS18561882 significantly suppresses PCa cell growth *in vitro* and *in vivo*.**

A. Cell proliferation was detected by cell counts in C4-2B cells treated with DS18561882 at indicated doses (0µM, 10µM, 50µM, 100µM). ****p* < 0.001. d, days.

B-D. Castrated mice possessing xenografts (VCaP-Vector and VCaP-PPFIA4) received vehicle control or DS18561882 treatment (100 mg/kg, n = 5/group, p.o.). Caliper measurements were taken twice every week to obtain tumor volume (B). Tumors were collected and weighed (C) after the mice were sacrificed. IHC staining of Ki67 on tumor slide from each group is shown (D). ***p* < 0.01, ****p* < 0.001. Scale bars, 20 µm.

E. Body weight of LNCaP and VCaP tumor-bearing mice in each group (n = 5/group).

F. Drug toxicity in vital organs in each group. The livers and kidneys were photographed and stained with hematoxylin-eosin in each group.
